# Establishment of the first Chinese national standard for recombinant nuclease used as a process material in pharmaceutical manufacturing

**DOI:** 10.3389/fbioe.2025.1695176

**Published:** 2025-12-02

**Authors:** Lyuyin Wang, Yue Sun, Kaixin Xu, Xinyue Hu, Yi Li, Ping Lyu, Xiao Liu, Jun Li, Jing Li

**Affiliations:** 1 National Institutes for Food and Drug Control, State Key Laboratory of Drug Regulatory Science, NHC Key Laboratory of Research on Quality and Standardization of Biotech Products, NMPA Key Laboratory for Quality Research and Evaluation of Biological Products, NMPA Key Laboratory for Quality Research and Evaluation of Chemical Drugs, Beijing, China; 2 Shanghai Yaxin Biotechnology Limited Company, Shanghai, China; 3 Sinovac Biotech Co., Ltd., Beijing, China

**Keywords:** recombinant nuclease, national standard, enzymatic activity assay, herring sperm DNA substrate, methodological validation, collaborative calibration

## Abstract

**Introduction:**

Recombinant nuclease (RSN) is a critical raw material enzyme essential for removing host nucleic acids during the production of cell and gene therapy products. However, the lack of standardized quality evaluation methods and reference materials for RSN has resulted in poor reliability and comparability of testing results, hindering the standardized development of related industries. This study aimed to establish the first Chinese national standard for RSN activity, thereby enabling accurate and comparable quantification of enzymatic potency across laboratories and industries.

**Methods:**

High-purity candidate RSN reference materials were prepared and structurally analyzed via mass spectrometry. A robust enzymatic activity assay using herring sperm DNA as the substrate was optimized and validated in accordance with ICH Q2 (R2) guidelines. The assay’s specificity, accuracy, and precision were evaluated, and a collaborative study involving three laboratories was subsequently conducted to determine the potency value.

**Results:**

The assay exhibited excellent precision (intra-lab coefficient of variation (CV) < 5%, inter-lab CV 2.01%) and accuracy (recovery rate 90–110%). The candidate reference material showed high homogeneity and stability. Collaborative testing confirmed the statistical consistency of the study results (P > 0.05), and the potency of the candidate material was determined to be 518 U/μL.

**Discussion:**

The establishment of this national standard will provide a unified reference for measuring RSN activity, enhance consistency in quality control, and support the safe development and production of biopharmaceuticals (including cell and gene therapies). This standard will serve as a critical tool for regulatory evaluation and industrial applications in China. The optimized content is consistent with the copyedited version of the article, featuring clear logic and comprehensive key information.

## 1 Introduction

The safety of biological products hinges on strict control over potentially hazardous impurities. Among these, residual host nucleic acids have emerged as a core indicator in global pharmaceutical regulation, given their potential to induce tumorigenicity, infectivity, and immune interference. Nucleic acid impurities may be generated during the production of vaccines, cell and gene therapy products, and recombinant protein drugs—spanning cell collection, viral vector construction, and final product purification. Therefore, the efficient removal of such impurities is pivotal to ensuring the clinical safety of these products. Nuclease (EC: 3.1.30.2), a non-specific endonuclease derived from *Serratia marcescens* (Eaves and Jeffries, 1963; Ball et al., 1987; Schwardmann et al., 2020), efficiently degrades DNA and RNA in all forms (e.g., double-stranded, single-stranded, linear, and circular), digesting them into oligonucleotides that are 2–5 bases in length (Biedermann et al., 1989; Lunin et al., 1997; Cheng et al., 2003). Recombinant nuclease (RSN) of non-animal origin, with its high purity, safety, stability, efficiency, broad spectrum, and ease of operation, has become the preferred tool for eliminating residual nucleic acids in biological products. In vaccine production, treating harvest fluids with RSN effectively reduces nucleic acid residues, enhances vaccine purity and stability, and ensures product quality and safety (Li et al., 2014; Kim et al., 2021; Moreira et al., 2021; Mayer et al., 2023; Ruan et al., 2023). In recombinant protein drug preparation, RSN lowers the nucleic acid content in fermentation broths, reduce solution viscosity, optimize downstream purification processes, and improve protein yield and quality (Biedermann et al., 1989; Yang et al., 2023). Within cell and gene therapy, RSN also plays an irreplaceable role in removing exogenous nucleic acids introduced during production, thereby safeguarding the safety and efficacy of therapeutic products (U.S. Department of Health and Human Services et al., 2020).

RSN is a recombinant endonuclease expressed in *Escherichia coli* via recombinant DNA technology. It is composed of 266 amino acids with two pairs of disulfide bonds and has a molecular weight of approximately 27 kDa. Despite its widespread application, RSN faces significant challenges in quality control and standardization. As it is not yet included in pharmacopoeias globally, manufacturers rely on in-house standards for production quality control, resulting in inconsistencies in enzymatic activity unit definitions, detection methods, and standard limits across commercial products. This not only complicates the uniform dosing of RSN—used as key process parameters during production (Liu et al., 2021; von Elling-Tammen et al., 2025)—but also increases the risk of residual host nucleic acids or nucleases in final products, creating hurdles for regulatory quality evaluation. In June 2025, the United States Pharmacopeia (USP) released the world’s first reference standard for RSN activity determination (The United States Pharmacopeial Convention, 2024), standardizing enzymatic activity assay methods and units. However, China has yet to establish a corresponding reference standard, leading to a lack of traceability for enzymatic activity values in production, quality control, application, and regulatory evaluation—severely impeding the standardized development of related industries. The demand for high-quality nucleases has surged with the rapid growth of emerging fields such as cell and gene therapy. Thus, establishing the first batch of national reference standards for RSN has become an urgent priority for China’s biopharmaceutical industry.

To this end, we prepared candidate RSN reference material samples. Structural characterization was performed using multiple analytical methods, while an enzymatic activity assay employing herring sperm DNA as a substrate was optimized and validated. We then comprehensively evaluated the candidate reference material in terms of activity, purity, protein content, homogeneity, and stability. Through a collaborative calibration process, the first Chinese national standard for RSN was established. This standard will provide a unified reference for quantifying RSN activity while ensuring measurement traceability, and it will also enhance quality control in biopharmaceutical production, particularly for cell and gene therapies. This work addresses a critical gap in China’s regulatory framework and promotes industrial standardization.

## 2 Materials and methods

### 2.1 Candidate reference material

The candidate RSN reference material (batch number: RSN240604) was provided by Shanghai Yaxin Biotechnology Co., Ltd. (Shanghai, China). It was prepared by expressing the endonuclease gene from *Serratia marcescens* in *E. coli* BL21 (DE3), followed by cultivation, bacterial harvesting, purification, sterilization, and the addition of 50% glycerol. The average filling volume was 100 μL per vial, with a coefficient of variation (CV) < 5%, indicating good filling uniformity.

### 2.2 Structural confirmation

#### 2.2.1 Relative molecular weight

High-resolution mass spectrometry (Xevo G2-XS QTof, Waters, Shanghai, China) was used to analyze the relative molecular weight of the candidate reference material.

#### 2.2.2 Peptide sequence coverage

A combined enzymatic hydrolysis strategy was employed, in which the candidate reference material was digested with trypsin (Promega), chymotrypsin (Sigma), and V8 protease Glu-C (Wako). The enzymatic hydrolysates were desalted and separated by high-performance liquid chromatography (Acquity I-Class, Waters), followed by liquid chromatography-tandem mass spectrometry (LC-MS/MS) analysis (Xevo G2-XS Q Tof, Waters). The raw data and theoretical sequence of the test sample were imported into the UNIFI software (Waters) for peptide coverage analysis.

#### 2.2.3 Isoelectric point

Horizontal isoelectric focusing electrophoresis was performed. An appropriate amounts of the candidate reference material was placed in 10-kD ultrafiltration centrifugal tubes (Merck) and ultrafiltered with water; this was repeated three times, resulting in a protein concentration of approximately 1.0 mg/mL. An aliquot of this solution was mixed with 10% ampholyte and methyl red reagent to prepare the sample solution. For electrophoresis, 2 μL each of pI 3–10 isoelectric point markers and the sample solution were added. A standard curve of pI vs. migration distance (Rf) was plotted with the pI values of the markers as the X-axis and the Rf as the Y-axis. The isoelectric point of the candidate reference material was calculated by substituting its migration distance into the standard curve.

#### 2.2.4 Maximum UV absorption wavelength

UV-visible spectrophotometry was used, with a 50% glycerol solution for baseline correction. An appropriate amounts of the candidate reference material was measured to determine their maximum absorption wavelength (λmax) in the range of 200–400 nm.

### 2.3 Purity

#### 2.3.1 Sodium dodecyl sulfate-polyacrylamide gel electrophoresis (SDS-PAGE)

An appropriate amount of the candidate reference material was diluted with water to prepare solutions of 1%, 0.5%, and 0.2%. Aliquots of the candidate reference material and its diluted solutions were mixed with 4× non-reducing loading buffer, heated in a boiling water bath at 100 °C for 5 min, cooled to room temperature, and centrifuged to obtain the sample solution and 1%, 0.5%, and 0.2% self-control solutions. A Novex WedgeWell 12% Tris-Glycine Gel was used, with 50 μL of each solution and 5 μL of Precision Plus Protein Standards (BIO-RAD) loaded into the wells. Electrophoresis was performed at a constant voltage of 100 V until bromophenol blue migrated to the bottom of the gel. The gel was fixed, stained with Coomassie brilliant blue, destained, photographed, and analyzed using a gel scanning system. The impurity limits were evaluated by comparing the staining intensity of any bands other than the main band in the sample electrophoresis profile with the main band of the 1% self-control solution.

#### 2.3.2 Size-exclusion chromatography (SEC-HPLC)

A TSKgel G2000SWXL column (7.8 mm × 30 cm) was used with a mobile phase of isopropanol−0.075 mol/L phosphate buffer (15:85) at a flow rate of 0.5 mL/min, column temperature of 28 °C, and detection wavelength of 214 nm. An appropriate amounts of the candidate reference material was mixed with an equal volume of the mobile phase to prepare the sample solution. A blank solution was prepared using 50% glycerol solution in the same manner. Subsequently, 50 μL of each solution was injected into the HPLC system, and the chromatograms were recorded. Purity was calculated using the peak area normalization method. The specificity of the method was evaluated by checking for obvious interference peaks in the blank solution chromatogram. For precision assessment, two analysts each tested three vials of the candidate reference material, and the relative standard deviation (CV) of the main peak retention time and peak area percentage was calculated. For stability testing, the candidate reference material was incubated at 25 °C, 40 °C, and 60 °C for 1 h and at 60 °C for 1, 2, and 3 h. The purity was determined as described above to observe changes in the main component and high-molecular-weight polymers.

### 2.4 Protein content

#### 2.4.1 Determination of experimental extinction coefficient using an amino acid analyzer

The RSN stock solution (batch number: NSY-RSN-400L-240601), internal standard amino acid (N-Val), and reference amino acids (Ser, Glu, Leu, Tyr, His, Arg, Pro) were hydrolyzed with 6N hydrochloric acid and 1% phenol solution. The amino acid composition of the stock solution was determined using an amino acid analyzer. Its molar concentration was calculated based on the theoretical number of amino acids, and the protein concentration was further calculated using the molecular weight determined in Section 2.2.1. The absorbance at 280 nm was measured as described in Section 2.4.2, and the experimental extinction coefficient of the RSN was calculated using the Lambert-Beer law (c = A/EL).

#### 2.4.2 Determination of protein content by UV spectrophotometry

The protein content of the candidate reference material was measured using the theoretical molar extinction coefficient of RSN at 280 nm (1.68, defined as the absorption coefficient of 1 mg/mL RSN at 280 nm). Additionally, the protein content was determined by UV-visible spectrophotometry using the experimental extinction coefficient obtained from the amino acid analyzer. The average value from the two extinction coefficients was taken as the protein concentration of the candidate reference material.

### 2.5 Enzymatic activity assay

To establish enzymatic activity, 6 mL of pre-cooled (2 °C–8 °C) substrate solution (herring sperm DNA diluted with an assay buffer [4 mM MgCl_2_ in 0.05 M Tris buffer, pH 8.0] to a final concentration of 0.1 mg/mL) was mixed with 300 μL of the candidate reference material solution (0.2 mg/mL candidate reference material diluted 30,000-fold with assay buffer). Next, 500 μL aliquots of this mixture were transferred to 12 DNase-free EP tubes, with three replicate tubes for each of the four reaction time points. For blank solutions, 6 mL of pre-cooled substrate solution was mixed with 300 μL of assay buffer (containing 0.1 mg/mL herring sperm DNA). The EP tubes were incubated at 37 °C for 15, 30, 45, and 60 min, before adding 500 μL of stop solution (4% perchloric acid), mixing, and placing on ice for 30 min. After pre-cooling, the samples were centrifuged at 4 °C for 7 min at 14,000 rpm. The supernatant was collected, and the absorbance at 260 nm was measured for both sample and blank solutions, with water as the reference.

Enzymatic activity was defined as the amount of enzyme that causes a change in A_260_ of 1.0 per 30 min at 37 °C and pH 8.0, denoted as 1 unit (U). The formula for calculation is as follows:

$$\text{Enzymatic activity }\left(U/\mu L\right)=\left(\Delta A\times 30\times V_{1}\times 2\times F\right)/\left(t\times V_{2}\times 1000\right)$$

where ΔA is the absorbance of the sample solution minus that of the blank solution at each time point; V_1_ is the total volume of the mixture, 6.3 mL; F is the dilution factor of the sample, 30,000; t is the incubation time of the mixture; V_2_ is the volume of the sample, 0.3 mL; 30 is the specified time for the defined enzymatic activity unit (30 min); 2 is the dilution factor from adding 500 μL stop solution to 500 μL reaction mixture; ΔA/t is the slope (a) of the linear regression equation y = ax + b, where y is ΔA and x is t; and 1,000 is the unit conversion from mL to μL.

### 2.6 Optimization of the enzymatic reaction conditions

#### 2.6.1 Detection principle

Purified DNA from herring sperm was used as the substrate. RSN acts on phosphodiester bonds in DNA molecules, degrading long double-stranded DNA into oligonucleotides or even mononucleotides. This disrupts the double-helical structure, weakens base stacking, and exposes more bases, increasing UV absorption at 260 nm. After adding perchloric acid to precipitate DNA fragments longer than 100 bp (terminating the reaction), the increase in absorbance at 260 nm in the supernatant was measured to determine nuclease activity (Nestle and Roberts, 1969; Friedhoff et al., 1996; Benedik and Strych, 1998).

#### 2.6.2 Quality release criteria for the substrate

Preliminary studies showed differences in activity when the candidate reference material was measured using herring sperm DNA substrates from three common brands (Invitrogen, Promega, and Solarbio), as described in Section 2.5. To investigate the cause, the molecular weights of the three substrates were analyzed by agarose gel electrophoresis. The ΔOD_260_ (OD_260_ at each time point minus blank OD_260_) was measured at 15, 30, 45, and 60 min using each substrate, and the slopes of the fitted linear equations (reaction rates) were compared by plotting ΔOD_260_ vs. time.

DNA substrates with larger and smaller molecular weights were used: after adding the candidate reference material, samples were taken at 5, 15, 30, 45, 60, and 90 min, and DNA fragments >100 bp were precipitated with perchloric acid. The precipitate was centrifuged, dissolved, and electrophoresed to observe changes in DNA molecular weight over time. The quality release criteria for the substrate were determined based on these results.

#### 2.6.3 Optimization of experimental parameters

Enzymatic activity determination relies on the maximum reaction rate to characterize the enzyme quantity; thus, the key conditions in the enzymatic system were optimized to approach the maximum reaction rate. Herring sperm DNA substrates were diluted to 0.002, 0.004, 0.006, 0.020, 0.040, and 0.080 mg/mL, and the enzymatic activity was measured as described in Section 2.5. A Michaelis-Menten plot (substrate concentration vs. enzyme activity) was generated to calculate the kinetic parameters Km and Vmax; the optimal substrate concentration was selected as > 10 × Km based on enzymatic kinetics theory.

The candidate reference material was diluted 10,000-, 15,000-, 20,000-, 30,000-, 40,000-, 60,000-, and 80,000-fold with assay buffer, and the detection range was determined by measuring the activity. Samples were taken every 15 min for 15–180 min to determine the optimal reaction time.

Assay buffers with different Mg^2+^ concentrations (0, 1, 2, 3, 4, 5, 10, 20, and 50 mM) were prepared to measure the activity and determine the optimal Mg^2+^ concentration. Buffers with different pH values (4, 5, 6, 7, 8, 9, and 10) were used to measure the activity and determine the optimal pH range. The candidate reference material was diluted in buffers (pH 4, 5, 6, 7, 7.5, 8, 8.5, 9, and 10) with or without 0.1 mg/mL bovine serum albumin (BSA), incubated at 25 °C and 37 °C for 2 h, and then assayed at pH 8.0 to evaluate the effect of BSA on activity and stability. The activity was measured at 25 °C, 30 °C, 37 °C, 42 °C, and 50 °C to determine the optimal temperature range. Dilutions of the candidate reference material were incubated at 25 °C, 37 °C, and 42 °C for 2 h and then assayed to evaluate stability within 60 min, confirming the final reaction temperature.

### 2.7 Method validation

In-house method validation was performed according to the International Council for Harmonisation of Technical Requirements for Pharmaceuticals for Human Use (ICH) Q2 (R2), including evaluations of specificity, linearity, precision (intermediate precision), accuracy, and range using the RSN candidate reference material.

#### 2.7.1 Specificity

The candidate reference material, 0.1 mg/mL BSA solution, and 0.2 mg/mL recombinant enterokinase (REK) solution were used to measure activity, as described in Section 2.5.

The candidate reference material was incubated at 25 °C, 40 °C, and 60 °C for 1 h and at 60 °C for 1, 2, and 3 h. The activity (Section 2.5) and purity (Section 2.3.2) were measured to evaluate interference from potential degradation products.

#### 2.7.2 Intermediate precision

The candidate reference material was diluted 20,000-, 24,000-, 30,000-, and 40,000-fold with assay buffer to prepare four samples with different activity levels (150%, 125%, 100%, and 75%). Two analysts measured each sample 16 times over 2 days, and the mean and CV% were calculated.

#### 2.7.3 Repeatability

One analyst prepared six replicate samples and assayed them in 1 day; the mean activity and CV% were calculated.

#### 2.7.4 Accuracy

The activity result from multi-laboratory collaborative calibration (Section 2.8) was used as the reference value. Accuracy was evaluated using the recovery rate (recovery% = measured activity/reference activity × 100%).

#### 2.7.5 Linearity and range

Linear regression analysis was performed using the reference reaction rates of samples at different levels as the X-axis and the corresponding measured values as the Y-axis.

### 2.8 Collaborative calibration

Collaborative calibration of the candidate reference material’s enzymatic activity was conducted by the National Institutes for Food and Drug Control, Shanghai Yaxin Biotechnology Co., Ltd., and Sinovac Life Sciences Co., Ltd. (randomly numbered Lab 1, Lab 2, and Lab 3). Each laboratory had two analysts performing two runs per day to obtain four valid results per day, totaling 20 results over 5 days. Data from the three laboratories were aggregated for precision and consistency analyses (Wang et al., 2023).

### 2.9 Evaluation of the homogeneity and stability of the candidate reference material

#### 2.9.1 Homogeneity assessment

The purity of 12 vials of the candidate reference material was determined by SEC-HPLC. The homogeneity was evaluated by calculating the CV% of the main peak retention time and area percentage across the 12 vials.

#### 2.9.2 Stability study

Two vials of the candidate reference material were stored at 4 °C for 5 and 10 days, and the SEC-HPLC purity and enzymatic activity were measured to evaluate accelerated stability. For long-term stability, enzymatic activity was measured at 0, 3, 6, 9, 12, 18, 24, and 36 months under −20 °C storage conditions.

## 3 Results

### 3.1 Structural confirmation of the candidate reference standard

#### 3.1.1 Relative molecular weight

**FIGURE 1:**
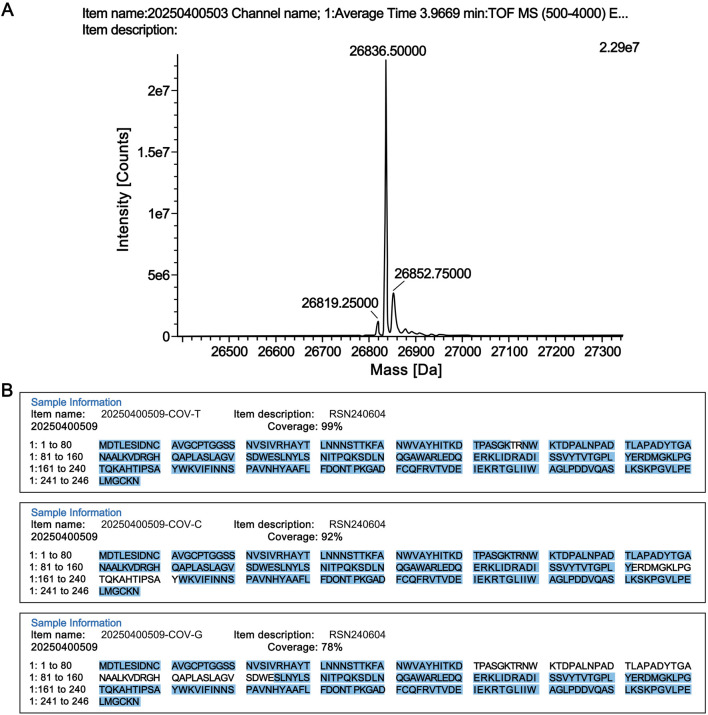
Structural confirmation of the candidate reference standard. **(A)** Molecular weight determination. **(B)** Peptide sequence coverage results of the candidate reference material digested by three proteases. COV-T: Trypsin; COV-C: Chymotrypsin; COV-G: Glu-C.

The molecular weight of the candidate reference standard was determined by high-resolution mass spectrometry. As shown in Figure 1A, the measured molecular weight was 26,836.50 Da, which is consistent with the theoretical molecular weight of 26,836.79 Da.

#### 3.1.2 Peptide sequence coverage

The RSN candidate reference material was digested with proteases, and the digested peptide samples were analyzed by liquid chromatography-tandem mass spectrometry (LC-MS/MS; Xevo G2-XS Q Tof, Waters). The LC-MS/MS data were further analyzed using UNIFI software (Waters), and the results confirmed that the product had 100% peptide sequence coverage (Figure 1B).

#### 3.1.3 Isoelectric point

Using the method described in Section 2.2.3, the isoelectric point of this batch of RSN candidate reference material was determined to be 6.7.

#### 3.1.4 Maximum UV absorption wavelength

Using the method described in Section 2.2.4, the maximum UV absorption wavelength (λmax) of this batch of the RSN candidate reference material was measured as 279.60 nm.

### 3.2 Purity

#### 3.2.1 SDS-PAGE

**FIGURE 2:**
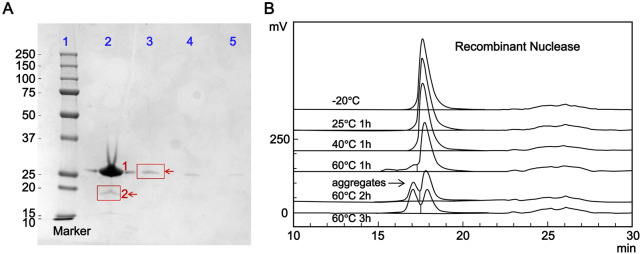
Purity analysis of the candidate reference standard. **(A)** Purity determination of the candidate reference material by sodium dodecyl sulfate-polyacrylamide gel electrophoresis. Lane 1: marker; Lane 2: candidate reference material (1: RSN, 2: impurity); Lane 3: 1.0% self-control solution; Lane 4: 0.5% self-control solution; Lane 5: 0.2% self-control solution. **(B)** Effect of different heating conditions on the purity of the candidate reference material evaluated by size-exclusion chromatography (SEC-HPLC).

The main component self-control method was used to prepare self-control solutions equivalent to 1.0%, 0.5%, and 0.2% of the candidate reference material concentration. Purity and impurity limit analyses were performed for these self-control solutions and the candidate reference material solution according to the method described in Section 2.3.1. The bands corresponding to 1.0%, 0.5%, and 0.2% of the candidate reference material concentration were all visible, confirming the method’s sufficient sensitivity (Figure 2A). The grayscale value of the impurity band with a molecular weight of approximately 19 kDa (10,056,624) in the candidate reference material lane (lane 2) was lower than that of the main component band in the 1.0% self-control solution lane (lane 3) (10,473,265), indicating that the purity of this batch of the candidate reference material was greater than 99.0%.

#### 3.2.2 Size-exclusion chromatography (SEC-HPLC)

Following the chromatographic conditions and method described in Section 2.3.2, 50 μL each of the blank solution and candidate reference material solution were injected into the HPLC system, and chromatograms were recorded. As shown in Figure 2B, no interference peaks were observed in the integration region of the main candidate reference material peak in the blank solution chromatogram, demonstrating the method’s good specificity. Two analysts separately determined the purity of three vials of the candidate reference material, and the results are shown in Table 1. The relative standard deviations (CV%) of the main peak retention time and peak area percentage were 0.03% and 0.12% (n = 6), respectively, confirming the accuracy of the method.

**TABLE 1:** Size-exclusion chromatography (SEC-HPLC) purity of RSN determined by two analysts (n = 6).

Test	Retention time (min)	Area (%)
Analyst 1–1	17.615	99.027
Analyst 1–2	17.615	99.265
Analyst 1–3	17.621	99.111
Analyst 2–1	17.616	99.213
Analyst 2–2	17.613	99.381
Analyst 2–3	17.611	99.220
Mean	17.615	99.203
CV%	0.003%	0.012%

The candidate reference material was subjected to heating at different temperatures and for different durations, with the untreated candidate reference material as a control, and its purity was evaluated. As shown in Figure 2B, the main peak area percentage remained above 99.0% (99.17%) after incubation at 25 °C for 1 h. After incubation at 40 °C for 1 h, the main peak area percentage dropped below 99.0% (98.97%). The purity decreased to 85.89% after incubation at 60 °C for 1 h, with a significant high-molecular-weight polymer peak (13.14%) appearing before the main peak. Further incubation at 60 °C for 2 and 3 h further decreased the purity to 63.49% and 50.75%, respectively, with the high-molecular-weight polymer content increasing to 35.08% and 47.82%, respectively. These results indicate that the proposed method can effectively detect heat-induced degradation products and reflect a decrease in purity.

### 3.3 Protein content

#### 3.3.1 Determination of experimental extinction coefficient using an amino acid analyzer

**FIGURE 3:**
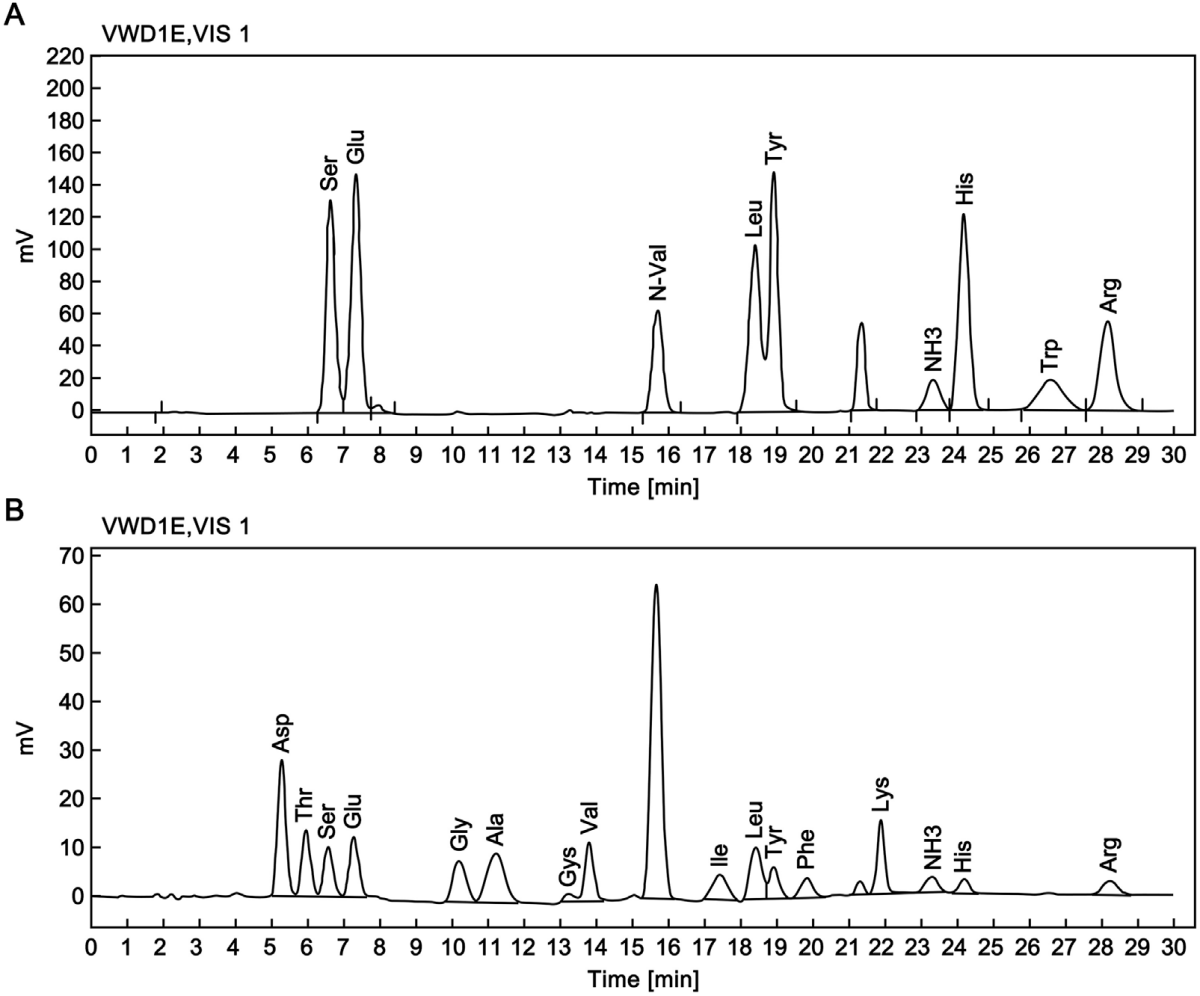
Amino acid composition analysis of the candidate reference standard. **(A)** Chromatogram of reference amino acid analysis. **(B)** Chromatogram of RSN amino acid analysis.

The RSN stock solution was subjected to acid hydrolysis. Accurate protein quantification was performed using the molar amount determined by an amino acid analyzer (Figure 3) and the molecular weight measured by mass spectrometry. Based on the Lambert-Beer law (c = A/EL), the experimental extinction coefficient (E_0.1_%) of the RSN was calculated to be 1.81.

#### 3.3.2 Determination of protein content by UV spectrophotometry

Using the theoretical extinction coefficient (E_0_._1_% = 1.68, defined as the absorption coefficient of 1 mg/mL RSN at 280 nm) calculated from the amino acid sequence of the candidate reference material, the protein content of 32 vials of the candidate reference material was determined using the method described in Section 2.4.2, with an average value of 0.20 mg/mL. Using the experimentally measured extinction coefficient (E_0_._1_% = 1.81), the protein content of the same 32 vials was calculated, with an average value of 0.19 mg/mL. The average of the two results (0.2 mg/mL) was taken as the protein content of the candidate reference material batch.

### 3.4 Optimization of enzymatic reaction conditions

#### 3.4.1 Quality release criteria for the substrate

**FIGURE 4:**
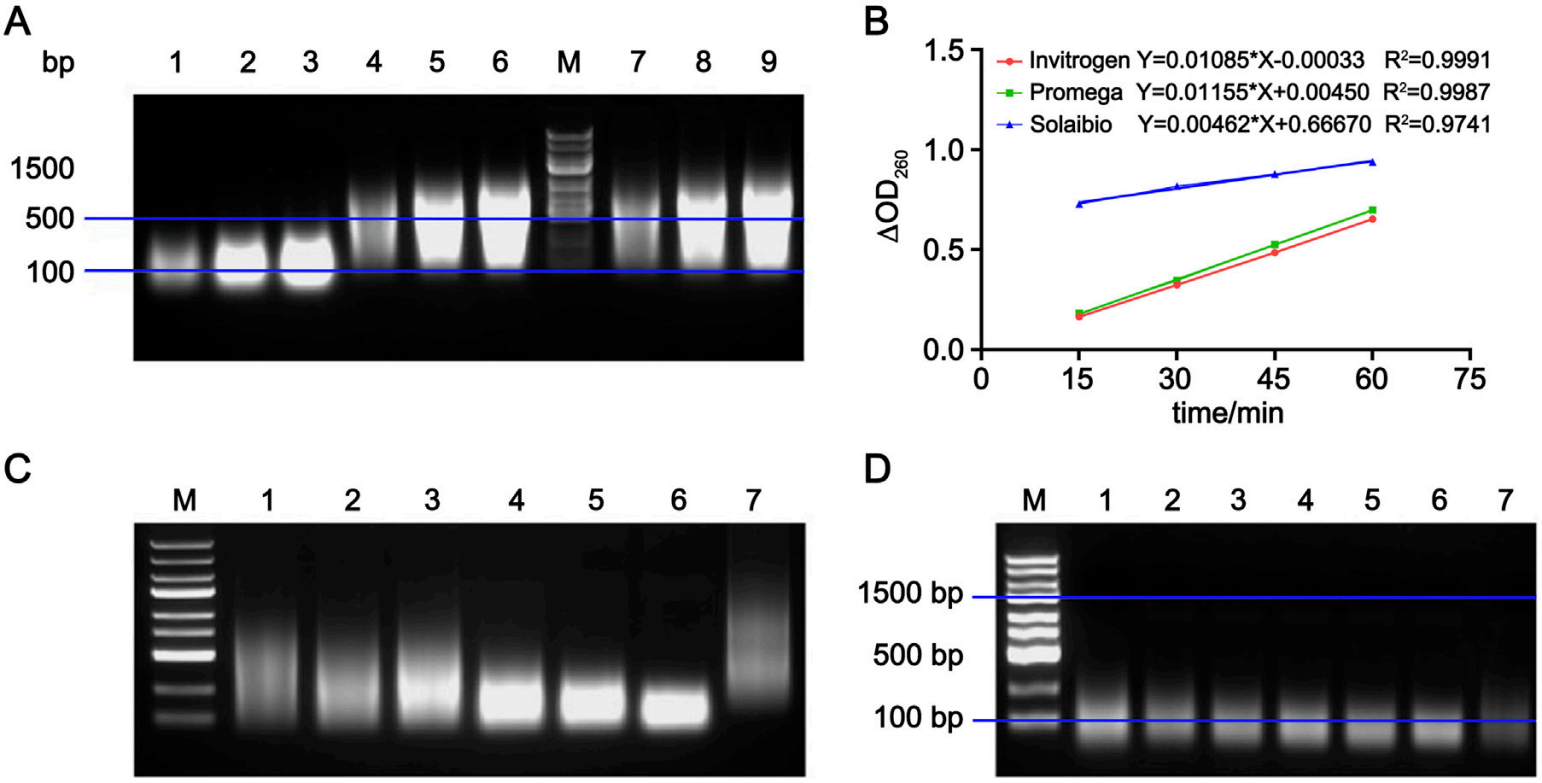
Establishment of substrate quality standards for the activity determination method. **(A)** Nucleic acid electrophoresis results of DNA substrates from three manufacturers. Lanes 1–3: DNA substrate from Solarbio; Lanes 4–6: DNA substrate from Invitrogen; Lanes 7–9: DNA substrate from Promega; M: marker. **(B)** Linear equations fitted by plotting ΔOD_260_ against time for reactions between RSN and DNA substrates from three manufacturers, n = 3. **(C)** Nucleic acid electrophoresis results of reactions between RSN and DNA substrate from Invitrogen at different times. Lanes 1–6: 5, 15, 30, 45, 60, and 90 min, respectively; Lane 7: unreacted DNA substrate control. **(D)** Nucleic acid electrophoresis results of reactions between RSN and DNA substrate from Solarbio at different times. Lanes 1–6: 5, 15, 30, 45, 60, and 90 min, respectively; Lane 7: unreacted control.

To investigate the cause of differences in the measured enzymatic activity of the candidate reference material when using different substrates, agarose gel electrophoresis was used to analyze the molecular weights of three herring sperm DNA substrates. As shown in Figure 4A, more than 75% of the DNA substrates from Invitrogen and Promega ranged from 100 to 1,500 bp, whereas the DNA substrate from Solarbio was concentrated in the 100–500 bp range. Using these three substrates, the ΔOD_260_ was measured at 15, 30, 45, and 60 min according to the method described in Section 2.5, and the ΔOD_260_ was plotted against time. As shown in Figure 4B, the slopes (k) of the linear plots (reaction rates) obtained with DNA substrates with higher molecular weights (from Invitrogen and Promega) were similar and more than twice that of the reaction rate measured with the low molecular weight substrate.

Diluted RSN candidate reference material was added to the DNA substrates from Invitrogen and Solarbio. Perchloric acid was added at 5, 15, 30, 45, 60, and 90 min, and the DNA precipitate was collected, dissolved, and electrophoresed. As shown in Figures 4C,D, the 100–1,500 bp DNA substrate from Invitrogen was gradually digested into 100–500 bp fragments with the extension of the enzymatic reaction time, while the low molecular weight (100–500 bp) DNA substrate from Solarbio showed no significant change in molecular weight. These results indicate that the greater the change in molecular weight of precipitated DNA over time, the larger the ΔOD_260_ in the supernatant, the higher the slope of the fitted line, and the faster the enzymatic reaction rate. Therefore, in this method, herring sperm DNA substrates with over 75% of fragments in the 100–1,500 bp range should be used.

#### 3.4.2 Optimization of experimental parameters

**FIGURE 5:**
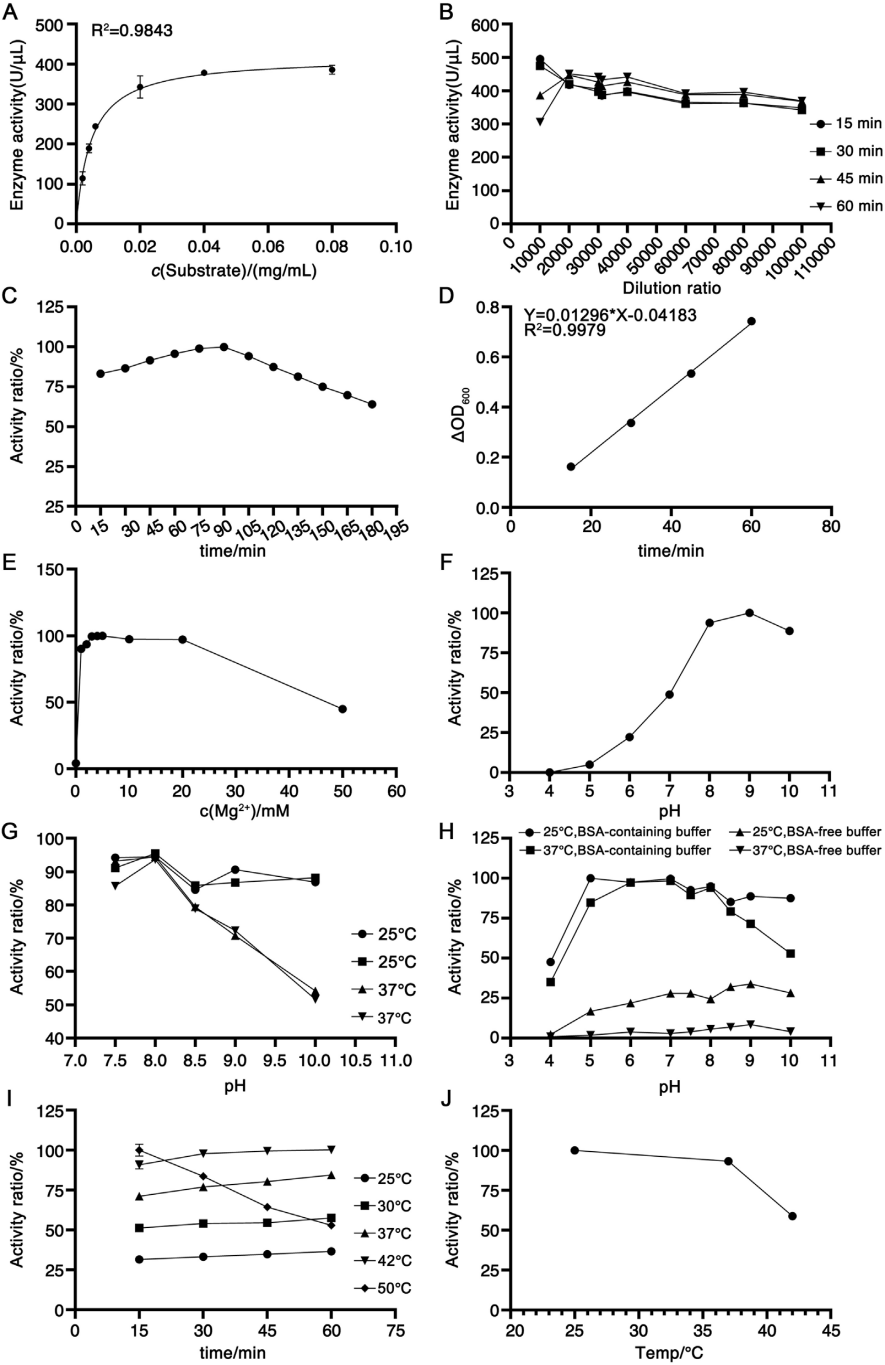
Optimization of experimental parameters for the newly established activity determination method. **(A)** Michaelis-Menten curve plotted with substrate concentration against RSN activity, n = 2. **(B)** Determination of candidate reference material activity at different dilution factors, n = 3. **(C)** Trend of candidate reference material activity changes with reaction time within 15–180 min, n = 3. **(D)** Linear equation fitted by plotting the ΔOD_260_ against time within 15–60 min, n = 3. **(E)** Effect of Mg^2+^ concentration on candidate reference material activity, n = 3. **(F)** Optimal pH of the assay buffer, n = 3. **(G)** Stability of the candidate reference material in assay buffers with different pH values, n = 3. **(H)** Effect of BSA on candidate reference material activity, n = 3. **(I)** Optimal enzymatic reaction temperature, n = 3. **(J)** Stability of the candidate reference material at different temperatures, n = 3.

The enzymatic kinetic parameters of the candidate reference material were determined using herring sperm DNA conforming to the aforementioned quality criteria as the substrate following the protocol described in Section 2.6.2. Based on the Michaelis-Menten equation, the calculated kinetic constants Km and Vmax were 0.0047 mg/mL and 417.6 U/μL, respectively. As illustrated in Figure 5A, at saturating substrate concentrations, the reaction rate approached Vmax and became independent of substrate concentration, exhibiting a linear correlation with the candidate reference material concentration. In accordance with enzymatic kinetic principles, the substrate concentration was selected to exceed 10×Km (i.e., 0.047 mg/mL), leading to the adoption of 0.1 mg/mL as the optimal substrate concentration. To define the detection range, the candidate reference material was serially diluted 10,000-, 15,000-, 20,000-, 30,000-, 40,000-, 60,000-, and 80,000-fold in assay buffer, with activity measured as detailed in Section 2.6.2. As shown in Figure 5B, the 10,000-fold dilution exhibited substantial variability across the 15-, 30-, 45-, and 60-min time points, whereas dilutions of 60,000-fold or higher resulted in diminished enzymatic activity. Consequently, the 20,000–40,000-fold dilution range was selected for subsequent analyses. For reaction time optimization, activity measurements were performed at 15-min intervals over 15–180 min. As shown in Figure 5C, the activity increased linearly within 15–90 min but declined after that. Within the 15–60-min window, linear regression of ΔOD_260_ against time yielded the equation Y = 0.01296X 0.04183 (R^2^ = 0.9979; Figure 5D), confirming robust linearity. Thus, this interval was designated as the optimal reaction period. Notably, the non-zero intercept of this regression precluded the use of the USP-recommended mean activity across time points; instead, the slope (ΔA/t) was substituted into the formula in Section 2.5 for activity calculation.

Given that RSN requires divalent cations for activity, with Mg^2+^ as a critical cofactor (Miller et al., 1999; Shlyapnikov et al., 2001), assay buffers containing 0–50 mM Mg^2+^ were evaluated. As shown in Figure 5E, maximal activity was observed at 4 mM Mg^2+^, with a gradual decline at concentrations exceeding 10 mM, leading to the selection of 4 mM Mg^2+^ in the assay buffer. The pH dependence of activity was assessed using buffers ranging from pH 4 to 10, revealing optimal activity at pH 8–9 (Figure 5F). To evaluate pH stability under assay conditions (room temperature dilution, 60-min incubation), the candidate reference material was diluted in buffers of pH 7.5, 8, 8.5, 9, and 10, followed by an incubation at 25 °C or 37 °C for 2 h. As shown in Figure 5G, pH 8 consistently yielded the highest activity and stability at both temperatures, thus defining the optimal pH. The impact of BSA was investigated by diluting the candidate reference material in pH 4–10 buffers with or without 0.1 mg/mL BSA, followed by 2-h incubation at 25 °C or 37 °C. As depicted in Figure 5H, BSA-containing buffers significantly enhanced activity, with maximal values at pH 8, leading to the use of BSA-supplemented pH 8 buffer. Temperature optimization revealed increased activity from 25 °C to 42 °C, with inactivation at 50 °C within 60 min (Figure 5I). Stability testing (2-h incubation at 25 °C, 37 °C, or 42 °C) demonstrated robust stability at 25 °C and 37 °C but marked inactivation at 42 °C (Figure 5J), resulting in the selection of 37 °C as the reaction temperature.

### 3.5 Methodological validation

#### 3.5.1 Specificity

No activity was detected for 0.1 mg/mL BSA solution or 0.2 mg/mL REK solution using this method, with relative activities of 0% and −0.9% compared to RSN, respectively.

**FIGURE 6:**
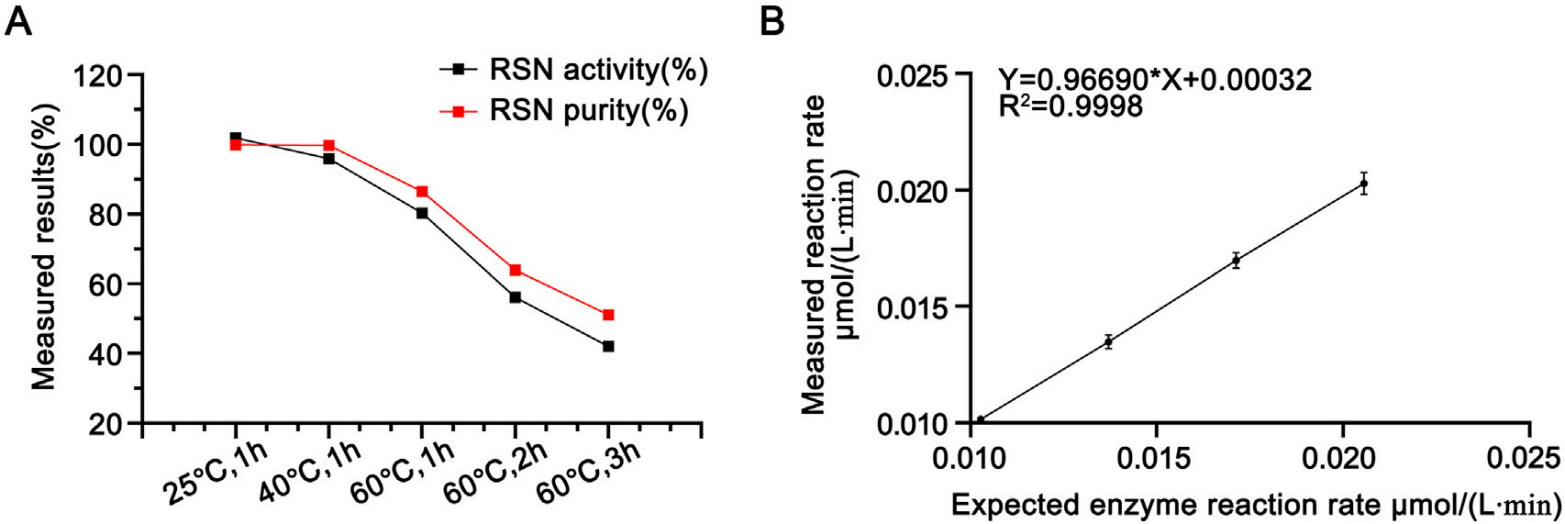
**(A)** Purity (determined by SEC-HPLC) and activity determination results of RSN with different heat treatments (with the enzymatic activity of untreated RSN set as 100%), n = 3. **(B)** Linearity of the newly established enzymatic activity determination method in this paper, n = 16.

Enzymatic activity was measured after incubating the candidate reference material at 25 °C, 40 °C, and 60 °C for 1 h, and the results are shown in Table 2. A paired t-test between the activity of the candidate reference material stored at −20 °C and that incubated at 25 °C for 1 h revealed no statistically significant difference (P = 0.1, >0.05). The RSN activity decreased with increasing temperature: incubation at 40 °C for 1 h resulted in approximately 10% activity loss, whereas incubation at 60 °C for 1 h led to ∼20% decrease. Extending the incubation time at 60 °C to 1–3 h further increased the activity loss from 20% to 60%, indicating that the method can reflect the reduction of RSN activity induced by heat. Size-exclusion chromatography (as described in Section 3.2.2) was used to analyze changes in the purity of the candidate reference material after incubation at 60 °C for 1, 2, and 3 h. As shown in Figure 6A, both activity and purity decreased gradually with prolonged heating, exhibiting consistent trends. This suggests that reduced purity is the primary cause of activity loss and confirms that the proposed method can effectively reflect heat-induced activity reduction.

**TABLE 2:** Enzymatic activity of heat-treated RSN.

Conditions	Test 1 (U/μL)	Test 2 (U/μL)	Test 3 (U/μL)
−20 °C	487.62	487.62	498.96
25 °C 1 h	514.08	495.18	491.40
40 °C 1 h	458.96	457.38	457.38
60 °C 1 h	396.90	396.90	389.34
60 °C 2 h	298.62	275.94	253.26
60 °C 3 h	181.44	207.90	230.58

#### 3.5.2 Intermediate precision

The candidate reference material was prepared into test solutions at four dilution levels (150%, 125%, 100%, and 75%), with 16 replicate measurements performed for each level. The results are presented in Table 3. The mean activities of the test solutions at the four dilution levels were 511.06, 513.25, 509.66, and 511.40 U/μL, with corresponding coefficients of variation of 2.99%, 3.38%, 3.47%, and 4.42%, respectively. All CV% values were less than 5%, indicating that the method showed good in-house intermediate precision.

**TABLE 3:** Enzymatic activity of RSN at different dilution levels (n = 16).

Concentration levels	Analyst 1 (U/μL)		Analyst 2 (U/μL)		Mean activity (U/μL)	CV%	Recovery%
	Day 1	Day 2	Day 1	Day 2			
150%	506.772	512.568	528.696	519.876	511.056	2.99%	98.67%
	517.356	492.660	505.008	511.560			
	523.656	491.400	499.968	532.476			
	527.436	495.684	483.588	528.192			
125%	538.272	501.984	478.094	534.946	513.248	3.38%	99.09%
	516.499	508.637	526.176	534.038			
	508.032	482.630	501.682	508.939			
	507.427	515.592	527.688	521.338			
100%	523.530	490.266	520.128	481.950	509.661	3.47%	98.40%
	530.712	518.616	514.080	485.730			
	537.138	521.640	522.774	494.424			
	491.002	518.238	489.888	514.458			
75%	495.432	530.712	473.261	538.776	511.403	4.42%	98.74%
	536.256	538.272	499.464	477.288			
	493.920	521.640	520.128	534.240			
	519.120	478.296	511.056	514.584			

#### 3.5.3 Repeatability

One analyst determined the enzymatic activity of six samples within a day, with an average value of 514.269 U/μL and a CV% of 2.59%, indicating good repeatability of the method in the laboratory.

#### 3.5.4 Accuracy

Taking the mean value of the collaborative calibration results under Section 3.6 as the reference value of the candidate reference material’s enzymatic activity (517.943 U/μL), the recoveries of the test solutions at the four dilution levels (150%, 125%, 100%, and 75%) in Table 2 were calculated to be 98.67%, 99.09%, 98.40%, and 98.74%, respectively, all within the range of 90%–110%, showing good accuracy of the method.

#### 3.5.5 Linearity and range

The reference values of the enzymatic reaction rates measured for the test solutions at each level were plotted against the corresponding determined values, and the results are shown in Figure 6B. The linear regression equation was Y = 0.96690X + 0.00032, with R^2^ = 0.9998, and the slope was close to 1.0, indicating that the method had good linearity within the dilution level range of 75%–150%.

### 3.6 Collaborative calibration

Three laboratories conducted collaborative calibration according to the method mentioned in Section 2.8, and the results are shown in Table 4. Each laboratory obtained four valid determination results per day, totaling 20 determination results over 5 days.

**TABLE 4:** Results of collaborative validation.

Day of the test	Number of tests	Lab 1 (U/μL)	Lab 2 (U/μL)	Lab 3 (U/μL)
Day 1	1	505.764	514.08	521.073
	2	537.138	521.64	523.152
	3	507.276	529.20	535.248
	4	507.276	514.08	517.671
Day 2	1	504.252	536.76	522.207
	2	523.908	514.08	524.475
	3	507.654	510.30	519.372
	4	518.238	502.74	520.317
Day 3	1	520.218	521.64	525.420
	2	502.362	514.08	527.688
	3	531.468	514.08	525.042
	4	519.372	514.08	532.602
Day 4	1	497.448	514.08	510.678
	2	500.850	510.30	516.159
	3	505.764	544.32	522.963
	4	514.458	529.20	515.403
Day 5	1	515.214	514.08	513.513
	2	514.080	517.86	512.379
	3	530.334	525.42	514.647
	4	515.214	521.64	514.647
Mean laboratory activity (U/μL)		513.914	519.183	520.733
CV% in the laboratories		2.11%	1.89%	1.27%
Mean activity between laboratories (U/μL)		517.943		
CV% between laboratories		1.85%		

#### 3.6.1 Precision analysis

The coefficient of variation (CV%) of the four daily detection results in each laboratory was calculated as the intra-day precision. A total of 15 intra-day precision values from three laboratories over 5 days were all less than 4%. The CV% of the mean detection results over 5 days in each laboratory was calculated as the inter-day precision, with three inter-day precision values from three laboratories being less than 2%. The CV% of detection results between two analysts in each laboratory was calculated as the inter-analyst precision, and all three inter-analyst precision values from three laboratories were less than 1%.

The CV% of 20 detection results in each laboratory was calculated as the intra-laboratory precision, which was 2.11%, 1.89%, and 1.27% for Laboratories 1–3, respectively, all being less than 5%. The CV% of 60 detection results across three laboratories was calculated as the inter-laboratory precision, which was 1.85%. These results indicate low variability within each laboratory and good reproducibility for the method between laboratories.

#### 3.6.2 Consistency analysis

**FIGURE 7:**
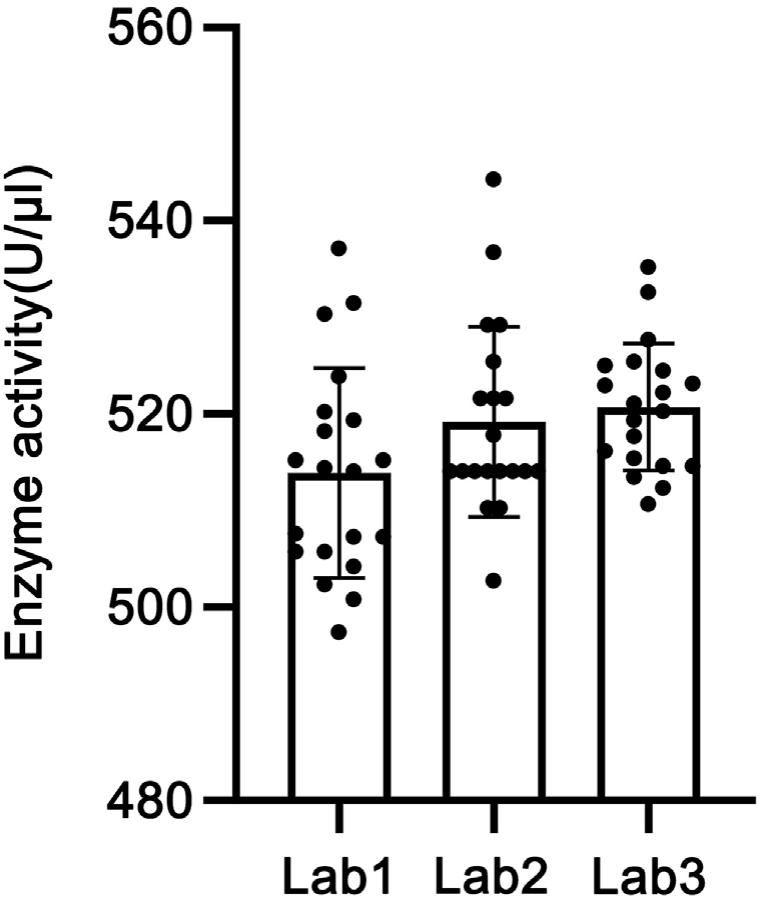
Results of the collaborative calibration of the candidate reference standard.

GraphPad Prism software was used to perform analysis of variance (ANOVA) on the enzymatic activity of the candidate reference material measured by the three laboratories. As shown in Figure 7, all three sets of data passed the D’Agostino & Pearson normality test (P > 0.05) and the homogeneity of variance test (P > 0.05). The results indicated no statistically significant difference in activity determination results among the three laboratories (P > 0.05), with statistical equivalence. Therefore, the mean value of the determination results from the three laboratories, 517.943 U/μL (rounded to 518 U/μL), was adopted as the activity of this batch of the candidate reference material.

### 3.7 Homogeneity and stability evaluation of the candidate reference standard

#### 3.7.1 Homogeneity assessment

Twelve vials of the candidate reference material were selected, and their purity was determined using the method described in Section 2.3.2. The CV% for the main peak area percentage of RSN was 0.14% (n = 12), and the CV% for the retention time was 0.03% (n = 12), indicating good homogeneity of the candidate reference material.

#### 3.7.2 Stability study

As shown in Table 5, three vials of the candidate reference material were stored at 4 °C for 5 or 10 days, and their activities were measured. Paired t-tests were performed to compare the mean activities with that of the candidate reference material stored at −20 °C, yielding P-values of 0.1183 and 0.9775, respectively, both of which were >0.05, indicating no significant differences. Size-exclusion chromatography was used to analyze the purity of the candidate reference material after storage at 4 °C for 5 and 10 days, with the main peak area percentages of RSN being 99.140% and 99.452%, respectively, both exceeding 99.0%. These results demonstrate that the candidate reference material remains stable when stored at 4 °C for 5 and 10 days.

**TABLE 5:** Stability results.

Conditions	Sample 1 (U/μL)	Sample 2 (U/μL)	Sample 3 (U/μL)	Mean value (U/μL)	CV%	P
−20 °C	498.96	487.62	487.62	487.62	1.3%	—
4 °C 5 days	483.84	480.06	487.62	483.84	0.8%	0.1181
4 °C 10 days	495.18	480.60	498.96	491.40	2.0%	0.9775

For long-term stability, the enzymatic activity of the candidate reference material remained above 80% after 18 months of storage at −20 °C, indicating that it can be stably stored at −20 °C for up to 18 months. In summary, the transportation and long-term storage conditions for this product are designated as −20 °C.

## 4 Discussion

During the production of cell and gene therapy products, RSN is dosed based on activity units as a critical process parameter. However, changes in production process conditions, enzyme formulation, and transportation may alter enzymatic activity. This increases the risk of inadequate control over residual host cell DNA or RSN (National Medical Products Administration and National Health Commission, 2025). Therefore, the accurate evaluation of RSN activity is crucial for ensuring the stability of the host cell DNA removal process and controlling the cost of enzyme usage. To address the current lack of standard activity determination methods and national reference standards for RSN in China, we have developed and established the first national reference standard for RSN. First, necessary structural studies were conducted on the candidate reference standard. Its structure was confirmed to be consistent with the theoretical value through multiple analyses. These included isoelectric point identification, maximum ultraviolet absorption wavelength identification, mass spectrometric molecular weight determination, and peptide map sequence coverage analysis. The purity determined by size-exclusion chromatography was greater than 99.0%, and the protein content was confirmed to be 0.2 mg/mL by ultraviolet-visible spectrophotometry and amino acid analysis. Homogeneity assessment results showed a CV% of only 0.14% in the main peak area across 12 candidate reference standards. This low variation fully meets the basic requirements for reference standards. On this basis, to further improve the accuracy and reliability of RSN activity determination results, the key parameters of the enzymatic reaction were systematically studied. Substrate quality standards were established through comparative studies of different herring sperm DNA substrates. A complete methodological validation was conducted in accordance with ICH Q2 (R2), and the results showed that the method had good specificity, precision (intra-laboratory CV < 5%), and accuracy (recovery rate 90%–110%).

Activity unit definition methods include both the newly established method and that published by the USP in June 2025, along with its first batch of recombinant nuclease reference standards. They calculate the activity by measuring the ΔA_260_ after RSN acts on the herring sperm DNA substrate at 37 °C for 15, 30, 45, and 60 min. The optimized method uses an activity determination buffer with an Mg^2+^ concentration of 4 mM for activity determination. Compared with the USP RSN activity determination method, which uses an activity determination buffer containing 1 mM Mg^2+^, the enzymatic activity is increased by more than 10%. In addition,the USP RSN activity determination method as the mean of results measured at four time points: 15, 30, 45, and 60 min. This approach is based on the key assumption that a linear fit of the measured values at these four time points against time should pass through the origin. However, experimental data demonstrate that the fitted equation exhibits a significant intercept, indicating the presence of background absorption in the blank system. To address this issue, our method utilizes the slope of the linear equation (ΔA/t) to calculate RSN activity, effectively solve the problem of large variation in the results measured at the four time points in the USP method (CV% is approximately 15%). When applying the absolute definition method for activity assignment of national standards, high precision is essential. We recommend adopting the slope calculation approach described in our method to ensure the accuracy and reliability of RSN activity determination results. In the optimization of enzymatic reaction conditions, the stability of the enzyme during the determination is a crucial but easily overlooked factor. In this study, we investigated the effects of buffer pH and temperature on the stability of RSN during the enzyme–substrate reaction. Finally, we selected an activity determination buffer containing BSA with pH = 8 for RSN activity determination at 37 °C. As an activity unit definition method, this method is susceptible to factors such as changes in reaction temperature, ionic strength and pH of the activity determination solution, time control, and operation by experimental personnel. In recent years, an increasing number of enzyme products have begun to adopt relative activity determination methods based on activity reference standards, which can overcome the influence of the above-mentioned factors and improve the accuracy and stability of detection results.

Using the newly established activity determination method to assign activity to the RSN candidate reference standard, we formulated a detailed collaborative calibration plan and invited three laboratories to jointly calibrate the activity of the candidate reference standard. Each laboratory conducted 20 independent determinations, and the intra-laboratory precision (CV% 1.27%–2.11%) and inter-laboratory precision (CV% 1.85%) of the detection results were good. No significant difference was found in the results of each laboratory (P > 0.05), showing statistical equivalence. Therefore, the total mean of the collaborative calibration, 518 U/μL, was taken as the activity of this batch of candidate reference standards. During collaborative calibration, we found that not setting the centrifuge temperature led to significantly higher detection results. This occurred when centrifuging after terminating the enzymatic reaction. After setting the centrifugation temperature to 4 °C, the determination results of the candidate reference standard were closer to those of the other two laboratories. Therefore, we further specified the centrifugation conditions after terminating the reaction by centrifugation at 14,000 rpm for 7 min at 4 °C after pre-cooling the centrifuge.

Next, stability studies were conducted to clarify the suitable transportation conditions for the candidate reference standard and exclude factors that may affect the characteristic values. The results showed that the purity and activity of the candidate reference standard did not significantly change after being placed at 4 °C for 5 and 10 days. Therefore, this product should be stored and transported at −20 °C. Long-term stability studies indicated that the candidate reference standard remains stable over extended use. In addition to being an indispensable tool enzyme in the production process of biological products, nuclease is also an impurity in the final product. Regulatory authorities around the world have pointed out that nuclease must be removed and its residual amount in the final product should be controlled. Currently, the enzyme-linked immunosorbent assay remains the most common method for detecting residual nuclease. This method is based on antigen-antibody binding. However, the accuracy of this method depends on the quality of the antibody and batch consistency, and it cannot evaluate the residual enzymatic activity. The national reference standard for RSN established in this study shows a good specific activity relationship, which may be applied to the quantitative detection of residual enzymatic activity, realizing the dual quantification of residual enzymatic activity and protein content.

For the first time in China, the RSN reference standard established in this study has achieved the unification of the characteristic value of the enzyme, namely, enzymatic activity. This effectively solves the problem of incompatibility of RSN activity units from different sources and provides a reliable basis for the precise feeding of RSNs in the production process. Moreover, it provides a standardized activity determination method and traceable standard for RSN manufacturers’ release inspection. This significantly improves the accuracy and comparability of detection results. More importantly, this reference standard has accumulated key data for the future inclusion of RSN general rules in the Chinese Pharmacopoeia, promoting the standardization process of recombinant nuclease and providing quality assurance for the industrial development of emerging fields such as cell and gene therapy.

## Data Availability

The original contributions presented in the study are included in the article/supplementary material, further inquiries can be directed to the corresponding author.
